# Obstetric complications in mothers with ADHD

**DOI:** 10.3389/frph.2022.1040824

**Published:** 2022-11-07

**Authors:** Caroline J. Walsh, Sofie L. Rosenberg, Elijah W. Hale

**Affiliations:** School of Medicine, University of Colorado Anschutz Medical Campus, Aurora, CO, United States

**Keywords:** ADHD, obstetrics, maternal ADHD, obstetric complication, comorbid ADHD

## Abstract

**Background:**

There is currently limited research on the intersection of pregnancy and ADHD and the unique pregnancy risk factors for mothers with an ADHD diagnosis. With an increased population of patients with ADHD in the recent decades and an increase in ADHD medication use during pregnancy it is important to consider what unique risks mothers with ADHD face during the perinatal period.

**Objective:**

Investigate a variety of outcomes in maternal ADHD.

**Methods:**

We identified female patients with a diagnosis of pregnancy and ADHD diagnosis. We also further separated the ADHD cohort for separate sub-analyses based on medication type. Odds ratios and relative risk were calculated from outcome incidence within each cohort. Cohorts were balanced on age, sex, and race.

**Results:**

We identified 45,737 pregnant females with ADHD. We matched these patients to pregnant females without ADHD, for a total of 42,916 pairs. Compared to the group without ADHD, mothers with ADHD had higher rates of every outcome except for HPV infection, which was statistically insignificant (*P* = 0.768). The odds ratios ranged from 1.08 for anemia complicating pregnancy to 2.63 for depressive episodes. Most outcomes were between 1.2 and 1.8 times more likely to occur in the cohort with ADHD.

**Conclusion:**

This study presents substantial advancements in our knowledge of pregnancy-related ADHD care. Armed with an increased awareness of these potential complications and their relationship with ADHD, obstetricians, psychiatrists, and providers of all specialties may be able to reduce the rate of complications within this specific patient population.

## Introduction

Attention-deficit hyperactivity disorder (ADHD) is one of the most common neurodevelopmental disorders, affecting an average of 5% of children globally and 2.5% of adults worldwide (1, 2). This estimate includes children diagnosed with ADHD that experience the persistence of symptoms into adulthood and ADHD diagnosed in adulthood. In recent decades, ADHD is increasingly recognized as a disorder affecting adults. Despite the prevalence of ADHD in adults, much of the research on childhood and adulthood ADHD has been done separately. Introducing a lifespan perspective of ADHD might improve understanding and management of the disorder (3).

Throughout the lifespan, ADHD is associated with several comorbidities and research consistently supports that those with ADHD are at greater risk of adverse life outcomes (4, 5). Comorbidities include depression, anxiety, and accidents (3). Early and appropriate recognition and diagnosis along with preventative care to mitigate these risks and treat other comorbidities associated with ADHD is important for children, adolescents, and adults with ADHD. There are significant barriers to care recognized for patients with ADHD. Barriers exist at many levels including identification of need and entry into care as well as factors such as older age, non-white ethnicity, rural residence, lower family socioeconomic status and female sex (1). Increasing knowledge about ADHD and how it presents throughout the lifespan among parents, primary care providers, and educators could improve access to care and reduce associated risk factors and comorbidities.

When considering ADHD with a lifespan perspective, one particularly important developmental period for clinicians to clearly understand associated health needs and risks is female adolescence. As physicians grow more comfortable assessing and diagnosing ADHD in children and adolescents, they can expect to see more women in their reproductive years with a history of ADHD. It is important to consider the risks associated with ADHD and the corresponding treatment or withheld treatment in the setting of pregnancy.

It is estimated that approximately 8% of women will experience some sort of pregnancy related complication (6). Complications of pregnancy and birth can include problems with blood pressure control, blood glucose levels, renal disease, anemia, infection, preterm birth, spontaneous abortion, still birth, thromboembolism, cardiac disease, thyroid disease, hyperemesis gravidarum, placenta previa, postpartum hemorrhage, and postpartum depression (7–21). While many obstetric complications are difficult to prevent, some have known methods to reduce the risk of their outcomes and mitigate the symptoms of disease during pregnancy. Gestational diabetes can impact up to 6% of pregnancies in the general population and 10%–20% in high risk groups (22). Complications of gestational diabetes include hypertensive disorders of pregnancy, polyhydramnios, and excessive fetal growth leading to malpresentation (10). Lifestyle modifications have been shown to greatly reduce the complications and severity of gestational diabetes. Such lifestyle modifications include following a close diet with high fruit and vegetable consumption and minimal processed foods, as well as physical activity before and during pregnancy (23).

Additionally, some obstetric complications require close monitoring and follow up, as there is no cure for these conditions, only management. Prime examples of these types of complications are the hypertensive disorders, specifically preeclampsia. 2%–5% of pregnancies are complicated by preeclampsia (24). It is recommended that women that are identified as high risk for preeclampsia should have early and preventative intervention as well as close surveillance (24). Outcomes of preeclampsia can impact both mother and child. For patients where preeclampsia worsens throughout pregnancy, an early delivery is often the only treatment. Many complications arise from premature delivery, including higher infant mortality and respiratory conditions (PPHN, RDS, TTN, and respiratory failure) (25). Outcomes of preeclampsia to the mother include end organ damage, HELLP syndrome, and mortality (26, 27). Overall, preeclampsia must be monitored closely to assess for the appropriate delivery time to minimize harm to both mother and infant (25–27).

There is currently limited research on the intersection of pregnancy and ADHD and the unique pregnancy risk factors for mothers with an ADHD diagnosis. There are many studies that investigate risk factors for children developing ADHD (maternal smoking, drug use, depression, parenting style), but even these studies rarely track the ADHD history of parents and do not consider the effect of ADHD on parental health (28). With an increased population of patients with ADHD in the recent decades and an increase in ADHD medication use during pregnancy it is important to consider what unique risks mothers with ADHD face during the perinatal period (29, 30).

While there is little data regarding the early infancy and pregnancy periods for women with ADHD, mental health issues such as depression, bipolar disorder, and anxiety disorders are associated with increased pregnancy and birth complications (31). Many of which are common comorbidities for individuals with ADHD, thus we might anticipate increased risks during pregnancy for individuals with ADHD. It is useful to consider how clinicians can safely treat mothers with ADHD while balancing the risks and benefits of treatment.

The gold standard treatment of ADHD is a combination of behavioral therapy and psychostimulant use, most often methylphenidate or amphetamine derivatives. Many women continue to need their medication throughout reproductive years and rely on their medication for optimal functioning in school, home, and the workplace (32). Prescriptions for ADHD medications during pregnancy are rising. A Canadian study showed that the absolute numbers of continuous use of psychotropic drugs during pregnancy, including ADHD medication, rose by the factor 6.4 between 2000 and 2011 (33). A Danish register-based study even suggested that the incidence of pregnancies exposed to ADHD medication increased more than 100-fold between 2003 and 2010 (34). The highest prevalence of ADHD medication use is often in the 3 months preceding conception and drops during the first trimester of pregnancy (29). During pregnancy, women that tend to be prescribed ADHD medication more often have other psychiatric disorders, mood and anxiety disorders (30). They are also more likely to be older, non-hispanic white, more highly educated, alcohol users during early pregnancy, nulliparous, and less likely to plan their pregnancies (29).

This is an especially important line of research as women with ADHD have an increased risk of unplanned pregnancies (35). Childhood ADHD is independently associated with becoming a teenage mother (36). Adolescents with ADHD are more likely to not only become pregnant at an earlier age but also have an overall greater incidence of pregnancy and are more likely to exhibit riskier sexual behaviors (37, 38). Studies suggest that long term treatment with ADHD medications decreases EP risk, pregnancy risk and reduces impulsivity and risky sexual behaviors (39, 40).

The aim of this study is to address pregnancy outcomes amongst patients with ADHD and those who do not have ADHD. Additionally, the obstetric outcomes of patients with ADHD will be further analyzed based on medication regimen.

## Methods

This study consisted of a population-based retrospective analysis of patient health records from the TriNetX database, which contains electronic medical records from large healthcare organizations and has shown utility for a variety of medical fields (6, 7). TriNetX, LLC is compliant with the Health Insurance Portability and Accountability Act (HIPAA), the US federal law which protects the privacy and security of healthcare data, and any additional data privacy regulations applicable to the contributing HCO (8). TriNetX is certified to the ISO 27001:2013 standard and maintains an Information Security Management System (ISMS) to ensure the protection of the healthcare data it has access to and to meet the requirements of the HIPAA Security Rule. Any data displayed on the TriNetX Platform in aggregate form, or any patient level data provided in a data set generated by the TriNetX Platform only contains de-identified data as per the de-identification standard defined in Section §164.514(a) of the HIPAA Privacy Rule (8).

Using International Classification of Diseases (ICD) codes, we identified all female patients with a diagnosis of pregnancy (Z33). We then separated patients by ADHD diagnosis (F90). We also further separated the ADHD cohort for separate sub-analyses based on medication type, including use of stimulant medications, non-stimulant medications, or either of these or no medication use. Stimulant medications were identified as belonging to either the amphetamine- or phenidate- classes. Non-stimulant medications included: guanfacine, clonidine, bupropion, atomoxetine, or viloxazine. Patients identified as on “any medications” were limited to the use of one identified medication at once. The 18 studied outcomes were identified from prior research on complication epidemiology. We studied pre-eclampsia, pre-existing hypertension, gestational hypertension, renal disease, post-partum depression, depressive episode, iron deficiency anemia, gestational diabetes, eclampsia, HPV, pre-term delivery, spontaneous abortion, cardiac disease complicating pregnancy, TORCH infections, anemia complicating pregnancy, malnutrition, early pregnancy hemorrhage, and hyperemesis gravidarum. Further information on specific codes and locations used can be found in Supplementary Table S2.

Using the TriNetX software, we performed 2 sets of statistical analysis. One analysis was between the cohort with ADHD and the cohort without, and the other analysis separated the ADHD cohort by medication type. Prior to comparison, the cohorts were balanced based on age, ethnicity, and race using nearest-neighbor matching to a difference in propensity scores <0.1. After matching, cohorts had no significant differences age, ethnicity, or race. Supplementary Table S1 contains cohort demographic information for the non-ADHD and overall ADHD cohorts, both before and after balancing on these characteristics. Balancing was performed before each of the analyses. As balancing can remove outliers, the control cohort for Table 2 does not have n's and risk percentages present, as the n would match the n of the comparison group. A *t*-test was used to compare event rates between cohorts. Relative risk and odds ratios with a 95% confidence interval were also calculated from event rates. The results from the overall analysis are compiled in Table 1, with the ADHD sub-analysis results compiled in Table 2. Significance for this study was set at *P* < 0.05. As this study contained only de-identified aggregate data, the Colorado Multiple Institutional Review Board (COMIRB) designated it as non-human research not in need of approval.

**Table 1:** Event statistics by cohort including cohort N, outcome N, absolute risk, relative risk, odds ratio with 95% CI, and *t*-test *P* value.

Event ICD 10	Cohort *N*	Event *N*	Risk	Odds Ratio	*P* value	Event ICD 10	Cohort *N*	Event *N*	Risk	Odds Ratio	*P* value
**Pre-eclampsia**						**HPV**					
ADHD	42,916	2,199	5.1%	1.300	**0** **.** **001**	ADHD	42,916	287	0.7%	1.025	**0** **.** **768**
No ADHD	42,916	1,712	4.0%	(1.218, 1.387)		No ADHD	42,916	280	0.7%	(0.869, 1.209)	
**Pre-existing hypertension**						**Preterm Birth**					
ADHD	42,916	1,365	3.2%	1.252	**0** **.** **001**	ADHD	42,916	1,213	2.8%	1.118	**0** **.** **008**
No ADHD	42,916	1,097	2.6%	(1.155, 1.357)		No ADHD	42,916	1,088	2.5%	(1.029, 1.215)	
**Gestational Hypertension**						**Spontaneous Abortion**					
ADHD	42,916	3,615	8.4%	1.327	**0** **.** **001**	ADHD	42,916	1,742	4.1%	1.250	**0** **.** **001**
No ADHD	42,916	2,782	6.5%	(1.261, 1.397)		No ADHD	42,916	1,405	3.3%	(1.164, 1.343)	
**Renal Disease**						**Cardiac Disease**					
ADHD	42,916	390	0.9%	1.312	**0** **.** **001**	ADHD	42,916	557	1.3%	1.299	**0** **.** **001**
No ADHD	42,916	298	0.7%	(1.127, 1.526)		No ADHD	42,916	430	1.0%	(1.145, 1.474)	
**Postpartum Depression**						**TORCH**					
ADHD	42,916	890	2.1%	1.800	**0** **.** **001**	ADHD	40,009	1,639	4.1%	1.514	**0** **.** **001**
No ADHD	42,916	499	1.2%	(1.612, 2.01)		No ADHD	41,155	1,129	2.7%	(1.402, 1.636)	
**Depressive Episode**						**Anemia**					
ADHD	22,963	3,604	15.7%	2.634	**0** **.** **001**	ADHD	42,916	2,239	5.2%	1.076	**0** **.** **019**
No ADHD	36,214	2,391	6.6%	(2.493, 2.781)		No ADHD	42,916	2,089	4.9%	(1.012, 1.144)	
**Anemia/Iron Deficiency**						**Malnutrition**					
ADHD	40,745	886	2.2%	1.276	**0** **.** **001**	ADHD	42,916	3,980	9.3%	1.211	**0** **.** **001**
No ADHD	41,758	715	1.7%	(1.155, 1.409)		No ADHD	42,916	3,341	7.8%	(1.154, 1.27)	
**Gestational Diabetes**						**Early Preg Hemorrhage**					
ADHD	42,916	2,045	4.8%	1.109	**0** **.** **002**	ADHD	42,916	1,830	4.3%	1.241	**0** **.** **001**
No ADHD	42,916	1,852	4.3%	(1.04, 1.183)		No ADHD	42,916	1,487	3.5%	(1.157, 1.331)	
**Eclampsia**						**Hyperemesis**					
ADHD	42,916	324	0.8%	1.577	**0** **.** **001**	ADHD	42,916	2,833	6.6%	1.581	**0** **.** **001**
No ADHD	42,916	206	0.5%	(1.324, 1.879)		No ADHD	42,916	1,836	4.3%	(1.489, 1.68)	

Bolded values indicate *P* < 0.05.

**Table 2:** Event statistics by medication type including cohort *N*, outcome *N*, absolute risk, relative risk, odds ratio with 95% CI, and *t*-test *P* value.

Event ICD 10	Cohort *N*	Event *N*	Risk	Odds Ratio	95% CI	*P* value	Event ICD 10	Cohort *N*	Event *N*	Risk	Odds Ratio	95% CI	*P* value
**Pre-eclampsia**							**HPV**						
**No Meds**	**Reference Cohort**						**No Meds**	**Reference Cohort**					
Any Meds	10,880	416	3.8%	0.727	(0.639, 0.828)	0.001	Any Meds	10,880	81	0.7%	1.504	(1.065, 2.124)	0.02
Stimulants	11,794	417	3.5%	0.684	(0.602, 0.777)	0.001	Stimulants	11,794	70	0.6%	1.251	(0.88, 1.78)	0.211
Non Stimulants	5,082	173	3.4%	0.670	(0.55, 0.816)	0.001	Non Stimulants	5,082	46	0.9%	1.488	(0.942, 2.351)	0.086
**Pre-existing Hypertension**							**Preterm Birth**						
**No Meds**	**Reference Cohort**						**No Meds**	**Reference Cohort**					
Any Meds	10,880	237	2.2%	0.668	(0.565, 0.789)	0.001	Any Meds	10,880	190	1.7%	0.585	(0.488, 0.701)	0.001
Stimulants	11,794	244	2.1%	0.649	(0.551, 0.764)	0.001	Stimulants	11,794	193	1.6%	0.546	(0.457, 0.652)	0.001
Non Stimulants	5,082	120	2.4%	0.699	(0.551, 0.886)	0.003	Non Stimulants	5,082	92	1.8%	0.598	(0.46, 0.777)	0.001
**Gestational Hypertension**							**Spontaneous Abortion**						
**No Meds**	**Reference Cohort**						**No Meds**	**Reference Cohort**					
Any Meds	10,880	686	6.3%	0.834	(0.751, 0.927)	0.001	Any Meds	10,880	300	2.8%	0.638	(0.55, 0.74)	0.001
Stimulants	11,794	754	6.4%	0.855	(0.773, 0.946)	0.002	Stimulants	11,794	344	2.9%	0.693	(0.602, 0.797)	0.001
Non Stimulants	5,082	331	6.5%	0.824	(0.709, 0.959)	0.012	Non Stimulants	5,082	160	3.1%	0.705	(0.573, 0.867)	0.001
**Renal Disease**							**Cardiac Disease**						
**No Meds**	**Reference Cohort**						**No Meds**	**Reference Cohort**					
Any Meds	10,880	58	0.5%	0.525	(0.381, 0.722)	0.001	Any Meds	10,880	87	0.8%	0.544	(0.418, 0.707)	0.001
Stimulants	11,794	77	0.7%	0.656	(0.491, 0.875)	0.004	Stimulants	11,794	113	1.0%	0.661	(0.521, 0.84)	0.001
Non Stimulants	5,082	20	0.4%	0.368	(0.22, 0.615)	0.001	Non Stimulants	5,082	40	0.8%	0.632	(0.424, 0.941)	0.023
**Postpartum Depression**							**TORCH**						
**No Meds**	**Reference Cohort**						**No Meds**	**Reference Cohort**					
Any Meds	10,880	208	1.9%	0.809	(0.672, 0.973)	0.024	Any Meds	9,884	332	3.4%	0.690	(0.599, 0.796)	0.001
Stimulants	11,794	202	1.7%	0.727	(0.605, 0.874)	0.001	Stimulants	10,696	310	2.9%	0.612	(0.531, 0.707)	0.001
Non Stimulants	5,082	113	2.2%	0.948	(0.731, 1.231)	0.69	Non Stimulants	4,543	156	3.4%	0.787	(0.636, 0.973)	0.027
**Depressive Episode**							**Anemia Complicating Pregnancy**						
**No Meds**	**Reference Cohort**						**No Meds**	**Reference Cohort**					
Any Meds	5,212	593	11.4%	0.681	(0.611, 0.759)	0.001	Any Meds	10,880	403	3.7%	0.621	(0.546, 0.705)	0.001
Stimulants	5,269	642	12.2%	0.733	(0.66, 0.813)	0.001	Stimulants	11,794	426	3.6%	0.587	(0.519, 0.664)	0.001
Non Stimulants	1,417	243	17.1%	1.009	(0.853, 1.193)	0.92	Non Stimulants	5,082	155	3.0%	0.565	(0.462, 0.691)	0.001
**Anemia/Iron Deficiency**							**Malnutrition**						
**No Meds**	**Reference Cohort**						**No Meds**	**Reference Cohort**					
Any Meds	10,190	175	1.7%	0.742	(0.609, 0.904)	0.003	Any Meds	10,880	508	4.7%	0.491	(0.44, 0.549)	0.001
Stimulants	11,043	182	1.6%	0.697	(0.576, 0.844)	0.001	Stimulants	11,794	584	5.0%	0.530	(0.477, 0.588)	0.001
Non Stimulants	4,655	102	2.2%	0.920	(0.701, 1.205)	0.543	Non Stimulants	5,082	187	3.7%	0.430	(0.36, 0.513)	0.001
**Gestational Diabetes**							**Early Pregnancy Hemorrhage**						
**No Meds**	**Reference Cohort**						**No Meds**	**Reference Cohort**					
Any Meds	10,880	342	3.1%	0.610	(0.531, 0.7)	0.001	Any Meds	10,880	311	2.9%	0.611	(0.529, 0.706)	0.001
Stimulants	11,794	361	3.1%	0.614	(0.537, 0.702)	0.001	Stimulants	11,794	343	2.9%	0.628	(0.547, 0.721)	0.001
Non Stimulants	5,082	141	2.8%	0.499	(0.406, 0.613)	0.001	Non Stimulants	5,082	111	2.2%	0.501	(0.397, 0.631)	0.001
**Eclampsia**							**Hyperemesis**						
**No Meds**	**Reference Cohort**						**No Meds**	**Reference Cohort**					
Any Meds	10,880	62	0.6%	0.665	(0.482, 0.918)	0.012	Any Meds	10,880	506	4.7%	0.606	(0.541, 0.68)	0.001
Stimulants	11,794	58	0.5%	0.602	(0.434, 0.835)	0.002	Stimulants	11,794	516	4.4%	0.572	(0.512, 0.64)	0.001
Non Stimulants	5,082	38	0.7%	0.926	(0.595, 1.443)	0.735	Non Stimulants	5,082	234	4.6%	0.688	(0.58, 0.817)	0.001

Bolded values indicate *P* < 0.05.

## Results

We identified 45,737 pregnant females with ADHD. We matched these patients to pregnant females without ADHD, for a total of 42,916 pairs with no statistical differences in age, race, or ethnicity. Compared to the group without ADHD, mothers with ADHD had higher rates of every outcome except for HPV infection, which was statistically insignificant (*P* = 0.768). The odds ratios ranged from 1.08 for anemia complicating pregnancy to 2.63 for depressive episodes. Most outcomes were between 1.2 and 1.8 times more likely to occur in the cohort with ADHD.

Sub-analysis of medication regimen revealed differences for each studied regimen. Compared to the cohort of ADHD patients without any recorded medications: patients on stimulant medications had statistically lower rates of every outcome, with the exception of HPV which was statistically insignificant (*P* = 0.211); patients on non-stimulant medications had lower rates in 13 of 18 outcomes, and 5 insignificant results; finally, patients on any individual medication had statistically significant results for all outcomes, which were lower for every outcome except for HPV.

**Figure 1:**
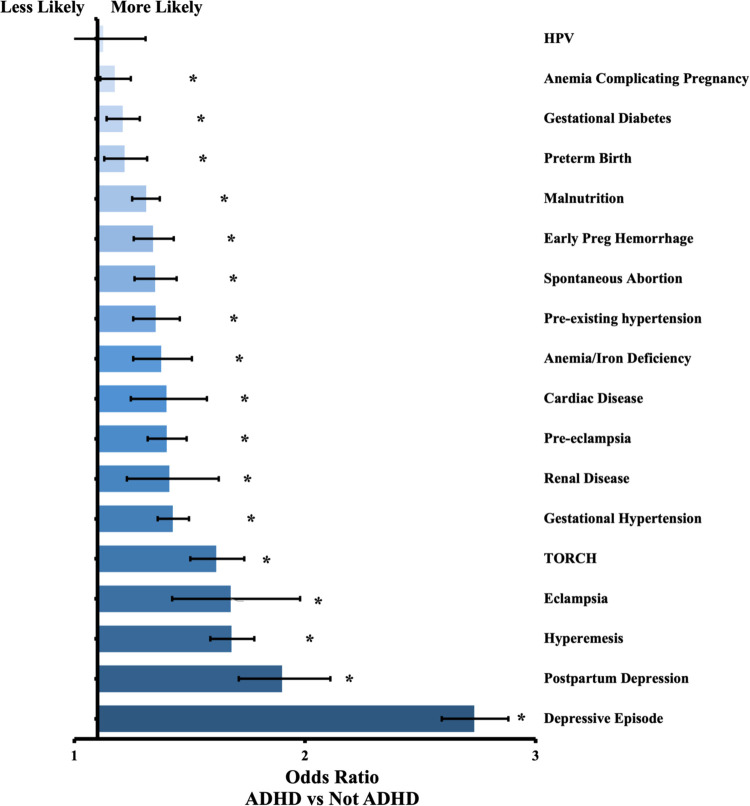
Odds ratio by outcome between patients with ADHD and without ADHD. Confidence bars represent 95% interval. * data labels indicate significant difference (*P* < 0.05).

**Figure 2:**
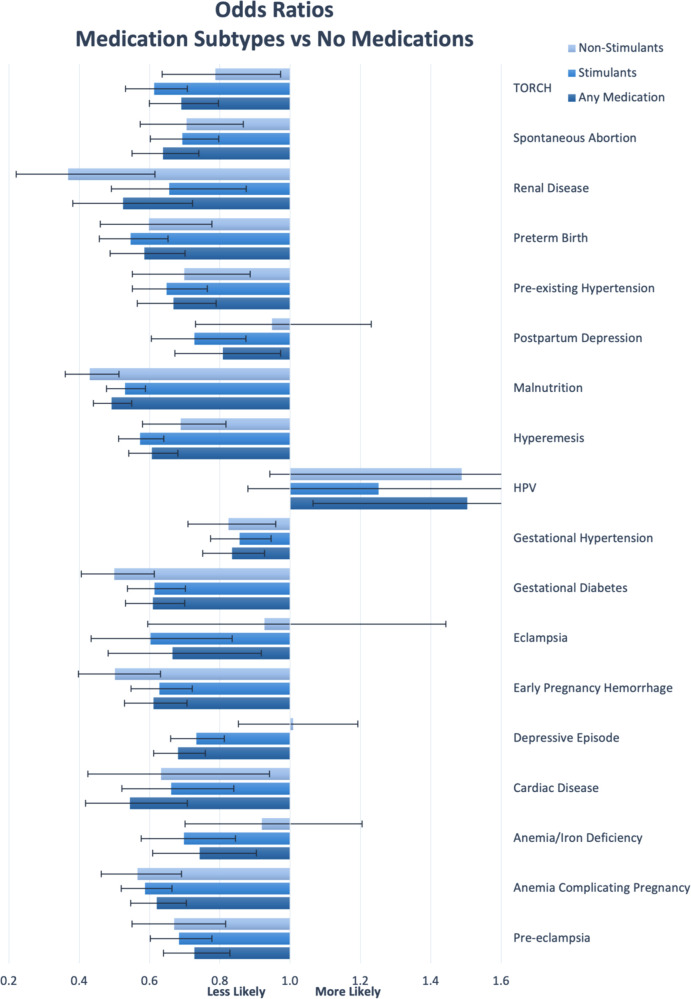
Odds ratio by outcome by medication type. Confidence bars represent 95% interval.

Complete results are in Tables 1, 2, along with odds ratios and confidence intervals. Figure 1 presents a visual depiction of the odds ratios between patients with and without ADHD. Figure 2 presents a visual depiction of odds ratios between each of the medication subtypes.

## Discussion

Our study confirmed higher rates of all studied outcomes in mothers with ADHD compared to those without ADHD. With the exception of HPV infection, statistical significance was present for all outcomes after adjusting for the potential confounding variables of age, race, and ethnicity. Our data aligned with prior areas of research for many outcomes, such as gestational diabetes and different presentations of hypertension (28). It also presented several novel findings that hold particular significance for comprehensive ADHD care. Specifically, the increased findings of hyperemesis gravidarum, anemia & iron-deficiency, TORCH infections, and both types of depression are relevant given known aspects of ADHD, but have yet to be studied in relation to pregnancy. In regard to hyperemesis gravidarum, ADHD is known to be associated with increased nausea, GI distress, and acid reflux (32). The underlying association may contribute to hyperemesis gravidarum, which is in turn connected with complications such as renal disease, anemia, and cardiac disease in pregnancy, as well as substantially higher rates of neurodevelopmental disorders like ADHD in the fetus (32, 40). TORCH infections, post-partum depression, and depressive episodes all relate to well-known sequelae of ADHD; patients with ADHD are more likely to engage in potentially risky behaviors, including unprotected sex, and are at significantly higher risk of developing depression than patients without ADHD (29, 30).

The two studied types of anemia, iron deficiency and nonspecific anemia complicating pregnancy, present an association that is a novel finding and a unique opportunity for beneficial intervention. Iron deficiency has recently become the focus of several studies relating to ADHD. Patients with ADHD have higher rates of iron deficiency, which can worsen on certain medications (41). Relatedly, ADHD symptomatology seems to worsen when patients develop iron deficiency anemia and improves when treated with iron supplements (34). Finally, ADHD and other neurodevelopmental disorders have also been shown to be associated with maternal iron deficiency during pregnancy (33). Our data show pregnant patients with ADHD have 1.28-fold higher rates of iron deficiency anemia. Given their ADHD may contribute to an increased likelihood to develop this deficiency, and the fact that iron deficiency may increase the likelihood of their fetus developing ADHD or another neurodevelopmental disorder like autism spectrum disorder (ASD), it may be worth increased awareness and caution regarding iron levels in pregnant women with ADHD. Providers could counsel their patients to be more conscious and active regarding adequate iron intake, or could schedule more regular laboratory screenings to prevent any baseline iron deficiency from progressing to anemia.

Our study is the first to present findings relating to multiple medication regimens and obstetric complications in ADHD. The first FDA approval for a non-stimulant drug was in 2004, and non-stimulant treatment of ADHD is becoming more common. Viloxazine was approved in 2021, and while bupropion has not been officially approved for ADHD use, we included it due to substantial research and its frequency of off-label use (42, 43). Interestingly, our group of patients on non-stimulants had the highest rate of statistically insignificant differences when compared to the reference group of patients without ADHD medication use. However, this may be due to the fact that non-stimulant medications are also frequently used for other conditions. Bupropion, atomoxetine, and viloxazine are all used to treat depression, and two of the five events lacking statistical significance were postpartum depression and depressive episode (42). Similarly, eclampsia also lacked statistical significance and involves hypertension, which both clonidine and guanfacine are approved to treat. Therefore, it is difficult to draw meaningful conclusions from the use of non-stimulant medications, as it is impossible to know if the medications were intended to treat ADHD, another condition, or if a discerning provider was intentionally choosing a medication to treat two conditions simultaneously.

With the exception of HPV, every outcome studied in the ADHD medication sub-analysis was less common for patients on medications compared to patients without medication use. Our methodology excluded patients using multiple ADHD medications at once, as the vast majority of ADHD patients do not require simultaneous use, and those that do typically have increased and therapy-resistant symptoms. For approximately half of the outcomes, non-stimulant medications had the largest reduction in risk. Cardiac disease complicating pregnancy was the only event with the “any medication” cohort having the largest reduction, with the remaining events having stimulant medications as the largest difference. The lack of a distinct pattern across risk differences between medication types may indicate the association present in our data is related to a conscious awareness of the patient's ADHD, rather than a specific health advantage of one subtype. For example, stimulant prescriptions are limited to a 3 months' supply in most states before the patient needs to interact with their provider again, yet non-stimulants are subject to different rules. Based on the widespread decrease in complication risk when a patient's ADHD is being treated with medications, the value of an ADHD-knowledgeable health care professional involved in a pregnant patient's care is substantial.

The safety of ADHD medication use during pregnancy has been well-studied. In a review of available data regarding perinatal exposure to psychostimulants and associated risks, researchers concluded that women with moderate-to-severe ADHD should not necessarily be counseled to suspend their ADHD treatment based on these findings (32). Untreated ADHD can lead to negative outcomes for both mother and infant. Studies have shown that pregnant women with ADHD may have greater difficulty with managing obstetric appointments which may increase the risk of negative health outcomes of undiagnosed and unmonitored complications such as preeclampsia and gestational diabetes (22, 24, 44). Since medications have been proven to help with the inattentive qualities of ADHD, it is important to consider these medications to be continued during pregnancy. Given the highly correlative nature of ADHD and other mental health conditions, one must consider comorbidities of untreated ADHD. Left untreated during pregnancy, individuals with ADHD might be at increased risk of depression, feelings of isolation, and familial conflict (45).

After reviewing the current literature, many ADHD medications are not shown to have significant negative outcomes in infant health and obstetric outcomes. Studies show that methylphenidate may have a slight increased risk of cardiac malformations, possible increased risk of spontaneous abortion, preterm birth, preeclampsia, and perinatal complications. However, methylphenidate is the preferred drug in the breastfeeding period and the risk of the above complications was quite minimal (46). Other studies have addressed the impact of amphetamines on infant health, but the majority study high doses of amphetamines associated with amphetamine abuse rather than doses prescribed in the management of ADHD (46). The data for ADHD level doses of amphetamines does not show an increased association with congenital malformations or impact on birthweight of the infant (47, 48). Medications less commonly prescribed for ADHD such as bupropion, atomoxetine, viloxazine, clonidine, and guanfacine do not have enough data to determine safety of use during pregnancy. Some studies showed concern that exposure to ADHD medication *in utero* may cause an increased risk of ADHD later in life. However, a recent study conducted by Lemelin showed that after adjusting for confounders there is no increased risk of *in utero* exposure to ADHD medications and increased risk of developing ADHD (49). While some medications have been shown to cause slight increases in negative fetal outcomes, other studies have suggested that birth outcomes may not be due to the medications themselves, but potentially underlying lifestyle factors or other comorbid diseases (41).

Our study has unique value for psychiatrists, obstetricians, and all providers treating pregnant patients with ADHD. While our study only focuses on association and does not ascribe causation to any one factor, the strength of the measured differences in risk suggests certain interventions may be worthwhile considerations. The first and most important intervention supported by our data is increased awareness and monitoring throughout the pregnancy of a female with ADHD. Many outcomes in our study can be prevented or mitigated through early intervention, and if providers have a higher index of suspicion for complications in patients with ADHD it may ultimately lower the prevalence of these conditions. Medication management of ADHD is another intervention that is supported by our data, given the nearly universal decrease in outcomes for patients on any of the studied medications. Finally, patients with ADHD may benefit from increased counseling from their health care providers, both before and during pregnancy. This counseling should address iron intake, safe sex practices, and the importance of regular prenatal and perinatal care. Visual and narrative information has shown particular utility in patients with neurodevelopmental disorders like ADHD. It may be worthwhile for providers to describe a case in which a patient did not receive adequate care in comparison to a case in which a patient did receive care that addressed both their pregnancy and their ADHD. If the provider is able to communicate effectively with the patient, the impact of ADHD on the pregnancy may be minimized.

While our study does address shortcomings in prior research of pregnancy and ADHD, such as controlling for age, race, and ethnicity, our study does have some limitations that may limit the generalizability of our findings. The database used for this study only contains deidentified, aggregate information, which prevents us from analyzing certain elements relevant to the field. For example, we are unable to know why patients were specifically prescribed non-stimulant medications that may have been used for a condition other than ADHD. Similarly, we are unable to confirm that patients were taking the recorded medications during their pregnancy. We also did not examine many other comorbid conditions, such as depression, anxiety, or substance use, which may have impacted the results. Finally, we limited our analysis to ICD codes, which likely underestimates the true rate of the complications, as ICD codes must be inputted manually. As such, it is unlikely providers would enter a code without the corresponding condition being present, but it is possible that a condition could be treated without the ICD code being recorded. However, the substantial size of our study mitigates each of these limitations and ensures our findings do have relevance for many patients and providers.

This study presents substantial advancements in our knowledge of pregnancy-related ADHD care. It contains a substantially larger patient base than previous research, and controls for age, race, and ethnicity; three confounding variables that can affect findings greatly. It also confirms associations that are known to affect patients with ADHD in general but have yet to be linked to maternal ADHD specifically, such as anemia and iron deficiency, hyperemesis gravidarum, TORCH infections, and depression. Armed with an increased awareness of these potential complications and their relationship with ADHD, obstetricians, psychiatrists, and providers of all specialties may be able to reduce the rate of complications within this specific patient population.

## Data Availability

The original contributions presented in the study are included in the article/**Supplementary Material**, further inquiries can be directed to the corresponding author/s.
