# Supplementary material for: Obstetric complications in mothers with ADHD
Source: Front Reprod Health. 2022 Nov 7;4:1040824. doi: 10.3389/frph.2022.1040824 (PMC9678343; doi:10.3389/frph.2022.1040824)
Supplement: Supplementary file 1 [file Table1.docx]

| Demographic | N | % of Cohort | *P*-value | Demographic | N | % of Cohort | *P*-value |
| --- | --- | --- | --- | --- | --- | --- | --- |
| (Pre-Matching) |  |  |  | (Post-Matching) |  |  |  |
| Current Age | | (Mean +/- SD) | | Current Age | | (Mean +/- SD) | |
| Not ADHD | 1,120,543 | 31.5 +/- 13.5 | < 0.001 | Not ADHD | 42,916 | 26.3 +/- 11.3 | 0.968 |
| ADHD - Overall | 45,737 | 26.3 +/- 11.3 |  | ADHD - Overall | 42,916 | 26.3 +/- 11.3 |  |
| White | | | | White | | | |
| Not ADHD | 585,133 | 59% | < 0.001 | Not ADHD | 32,815 | 76% | 0.754 |
| ADHD - Overall | 32,776 | 76% |  | ADHD - Overall | 32,776 | 76% |  |
| Black | | | | Black | | | |
| Not ADHD | 204,511 | 21% | < 0.001 | Not ADHD | 5,720 | 13% | 0.741 |
| ADHD - Overall | 5,753 | 13% |  | ADHD - Overall | 5,753 | 13% |  |
| Hispanic/Latino | | | | Hispanic/Latino | | | |
| Not ADHD | 150,051 | 15% | < 0.001 | Not ADHD | 2,511 | 6% | 0.569 |
| ADHD - Overall | 2,472 | 6% |  | ADHD - Overall | 2,472 | 6% |  |
| Not Hispanic/Latino | | | | Not Hispanic/Latino | | | |
| Not ADHD | 666,805 | 67% | < 0.001 | Not ADHD | 35,924 | 84% | 1 |
| ADHD - Overall | 35,924 | 84% |  | ADHD - Overall | 35,924 | 84% |  |

**Supplemental Table 1. Demographics of Overall ADHD cohort and Not ADHD reference cohort, before and after propensity score matching on Ethnicity, Race, and Age.**
