# Supplementary material for: Obstetric complications in mothers with ADHD
Source: Front Reprod Health. 2022 Nov 7;4:1040824. doi: 10.3389/frph.2022.1040824 (PMC9678343; doi:10.3389/frph.2022.1040824)
Supplement: Supplementary file 2 [file Table2.docx]

| Anemia Complicating Pregnancy | UMLS:ICD10CM:O99.01 |
| --- | --- |
| Anemia / Iron deficiency | UMLS:ICD10CM:E61.1 |
|  | UMLS:ICD10CM:D50 |
| Cardiac Disease | UMLS:ICD10CM:O99.41 |
| Depressive episode | UMLS:ICD10CM:F32 |
| Early Pregnancy Hemorrhage | UMLS:ICD10CM:O20 |
| Eclampsia | UMLS:ICD10CM:O15 |
| Gestational Diabetes | UMLS:ICD10CM:O24.4 |
| Gestational Hypertension | UMLS:ICD10CM:O13 |
| HPV | UMLS:LNC:17399-7 |
|  | UMLS:LNC:17401-1 |
|  | UMLS:LNC:44550-2 |
|  | UMLS:LNC:30167-1 |
|  | UMLS:LNC:71431-1 |
| TORCH | UMLS:ICD10CM:B58 |
|  | UMLS:ICD10CM:A51 |
|  | UMLS:ICD10CM:A52 |
|  | UMLS:ICD10CM:A53 |
|  | UMLS:ICD10CM:B16 |
|  | UMLS:ICD10CM:B18.0 |
| Hyperemesis | UMLS:ICD10CM:O21 |
| Malnutrition | UMLS:ICD10CM:O25 |
| Obstetric Embolism | UMLS:ICD10CM:O88 |
| Postpartum depression | UMLS:ICD10CM:F53.0 |
| Pre-eclampsia | UMLS:ICD10CM:O14 |
| Pre-existing Hypertension | UMLS:ICD10CM:O10 |
| Preterm Birth | UMLS:ICD10CM:O60.1 |
| Renal Disease | UMLS:ICD10CM:O26.83 |
| Spontaneous Abortion | UMLS:ICD10CM:O03 |
